# Clinical and diagnostic imaging findings in police working dogs referred for hip osteoarthritis

**DOI:** 10.1186/s12917-020-02647-2

**Published:** 2020-11-07

**Authors:** J. C. Alves, A. Santos, P. Jorge, C. Lavrador, L. Miguel Carreira

**Affiliations:** 1 Divisão de Medicina Veterinária, Guarda Nacional Republicana (GNR), Rua Presidente Arriaga, 9, 1200-771 Lisbon, Portugal; 2 MED – Mediterranean Institute for Agriculture, Environment and Development, Instituto de Investigação e Formação Avançada, Universidade de Évora , Pólo da Mitra, Ap. 94, 7006-554 Évora, Portugal; 3 Faculty of Veterinary Medicine, University of Lisbon (FMV/ULisboa), Lisbon, Portugal; 4 Interdisciplinary Centre for Research in Animal Health (CIISA), University of Lisbon, FMV/ULisboa, Lisbon, Portugal; 5 Anjos of Assis Veterinary Medicine Centre (CMVAA), Barreiro, Portugal

**Keywords:** Dog, Osteoarthritis, Hip, Stance Analysis, Digital Thermography Goniometry, Digital radiography, Clinical Metrology Instruments

## Abstract

**Background:**

Osteoarthritis (OA) is the most commonly diagnosed joint disease in veterinary medicine, with at least 80% of the cases of lameness and joint diseases in companion animals being classified as OA. Sporting and working animals are more predisposed to develop OA since they are exposed to chronic fatigue injuries, leading to bone and muscular tissue damage and failure, resulting in clinical signs. To characterize the clinical signs and diagnostic findings of Police working dogs presenting with bilateral hip OA at the time of diagnosis. Fifty animals were evaluated with a bodyweight ≥ 15 kg, be older than two years, and without any medication or nutritional supplements for ≥ 6 weeks.

**Results:**

Weight distribution, joint range of motion at flexion and extension, thigh girth, digital thermography, and radiographic signs were collected. Data from different Clinical Metrology Instruments (CMI) were collected: Canine Brief Pain Inventory, Liverpool Osteoarthritis in Dogs, Canine Orthopedic Index, and the Hudson Visual Analogue Scale. Results were compared by breed, age, sex, and Orthopaedic Foundation for Animals hip grades with the Independent Samples T-Test, ANOVA followed by a Bonferroni post hoc test, and Pearson correlation coefficient, with p < 0.05. The sample included 30 males and 20 females, with a mean age of 6.5 ± 2.4 years and a bodyweight of 26.7 ± 5.2 kg. Animals with weight distribution below normal levels had significant variations of joint extension and function scores. This evaluation was the only not correlated with at least one breed. Animals with caudolateral curvilinear osteophyte showed a poorer clinical presentation and worse scores in all considered CMIs. Radiographic changes correlated with age and corresponded to worse CMIs scores and weight distribution. Dutch Shepherd Dogs showed better CMI scores than the other considered breeds.

**Conclusions:**

Police working dogs presented with complaints related to hip OA at an early stage of the disease. Hip scores influenced clinical presentation, with moderate cases showing lower thigh girth and worse pain interference and severity, and function scores than mild cases. Patients with severe OA had lower thermographic evaluations than patients with moderate OA. Age was the primary variable influencing considered CMI scores.

## Background

Osteoarthritis (OA) is the most commonly diagnosed joint disease in both human and veterinary medicine, with at least 80% of the cases of lameness and joint conditions in companion animals being classified as OA [1–3]. Risk factors include breed, neutering, higher body weight, and being older than eight years [4]. Police and working animals are at increased risk of developing an orthopaedic disease than companion animals, and OA is common amongst these animals [5]. Hip OA is commonly bilateral and a consequence of canine hip dysplasia, being influenced by many genes specific for every breed [6–9].

Pelvic radiographs are frequently performed in dogs to screen hip dysplasia and OA. They have been used for over four decades in several screening mechanisms worldwide. They are also a significant determination of clinical and experimental outcome [10–12]. The most common radiographic view is the ventrodorsal hip extended view. The ventrodorsal flexed view (also called frog-legged view) enhances the visibility of the cranial and caudal aspects of the femoral head and neck. This feature helps assess the presence of circumferential femoral head osteophyte (CFHO) and caudolateral curvilinear osteophyte (CCO). These two features represent early radiographic signs that predict the development of the clinical signs of hip OA [9, 13–15].

Weight distribution and off-loading or limb favouring at stance is a commonly used subjective assessment during orthopaedic examination [16]. Animals with OA may not be overtly lame at a walk or a trot but exhibit subtle shifts in body weight distribution at a stance due to pain or instability [17, 18]. Stance analysis has been reported as sensitive for detecting lameness in dogs, with better results in large breed dogs [19]. Digital thermal imaging is a non-invasive, non-radiating, contact-free, physiologic diagnostic tool that depends on heat resulting from physiologic functions related to skin temperature control [20–22]. It has been described as useful in several species, from humans to horses and cats, but its clinical utility has rarely been studied in small animals [21, 23, 24]. Animals with OA present a variety of clinical signs, which can vary significantly. Muscular atrophy is a consistent finding and is evident within a few weeks of OA onset [8, 25]. Restricted range of motion (ROM), including flexion and extension, is usually present [8]. The evaluation of asymmetry, assessment of muscle atrophy level, measurement of static weight-bearing, and ROM measurement have been described as the most valid and sensitive physiotherapeutic evaluation methods [26, 27].

Pain and functional ability are also important parameters in the evaluation of OA treatment efficacy [28]. Pain is a multi-dimensional experience with sensory, evaluative, and affective components [29]. Several clinical metrology instruments (CMI) have been developed to measure outcome assessments to approach these different dimensions. In dogs, CMIs are typically completed by a proxy. The ones developed and validated for dogs are the Canine Brief Pain Inventory (CBPI) and the Liverpool Osteoarthritis in Dogs (LOAD) [30–33]. The CBPI allows to rate a dog’s pain and is divided into two sections, a pain severity score (PSS) that assesses the magnitude of the animal pain, and a pain interference score (PIS) that evaluates the degree to which pain affects daily activities [34]. The Canine Orthopaedic Index (COI) was developed for clinical research in canine orthopedics or individual outcomes in four domains: stiffness, gait, function, and quality of life. It has been shown to have excellent reliability and validity [35]. The Hudson Visual Analogue Scale (HVAS) has been deemed repeatable and valid to assess the degree of mild to moderate lameness in dogs, compared with force plate analysis as a criterion-referenced standard [36]. By collecting information from different CMIs, it possible to characterize the disease in all dimensions, a patient’s level of pain, the degree of lameness, the ability to enjoy life, and perform daily activities. It also allows characterizing the effect of a treatment in each of those dimensions.

This study aimed to characterize the clinical signs and diagnostic findings of Police working dogs presenting with bilateral hip OA. We hypothesized that differences occur when comparing breeds commonly used as Police working dogs.

## Results

The sample included 50 Police working dogs, of both genders (all intact, 30 males and 20 females), with a mean age of 6.5 ± 2.4 years, bodyweight of 26.7 ± 5.2 kg, and a body condition score of 4 (70%) or 5/9 (30%). Four breeds were represented: German Shepherd Dogs (GSD, *n* = 17), Belgian Malinois Shepherd Dogs (BM, *n* = 15), Labrador Retriever (LR, *n* = 10), and Dutch Shepherd Dog (DSD, *n* = 8). Fifteen patients did not meet the inclusion criteria.

Considering OFA hip grading, 35 animals were classified as mild (70%), 10 as moderate (20%), and 5 as severe (10%). Comparing animals classified as mild and moderate, significant differences were observed in thigh girth (*p* = 0.01), frequency of CCO in the frog-legged view (*p* < 0.01), and scores of PIS (*p* = 0.01), PSS (*p* = 0.02) and Function (*p* = 0.01), with moderate cases presenting worse evaluations. With digital thermography, significant differences were observed comparing moderate and severe OA in the dorsoventral (*p* = 0.03, 25.0 ± 1.8 and 24.0 ± 1.7, respectively) and lateral views (*p* = 0.04, 26.1 ± 2.5 and 25.5 ± 2.4, respectively).

Measured values of overall age, body weight, weight distribution, digital thermography, thigh girth, and joint range of motion, and divided by breed and sex, are presented in Table 1. Comparing males to females, significant differences were observed in weight and thigh girth (*p* < 0.01), with male dogs having higher values. Comparing breeds, GSD were significantly heavier than BM (*p* < 0.01) and LR (*p* < 0.01) and also had significantly higher thigh girth than BM (*p* < 0.01), LR (*p* < 0.01), and DSD (*p* = 0.02). LR were significantly older and had lower thigh girth than GSD (*p* < 0.01 for both), BM (*p* < 0.01 and *p* = 0.05, respectively), and DSD (*p* < 0.01 for both). DSD were significantly heavier than BM (*p* < 0.01). DSD also had higher measured values with digital thermography on the dorsoventral view than GSD (*p* = 0.02 for both) and on the lateral view than BM (*p* = 0.04). Thigh girth showed a correlation with breed (*r*=-0.34, *p* < 0.01), weight (*r*=-0.47, *p* < 0.01) and sex (*r*=-0.72, *p* < 0.01). Age correlated with joint extension (*r*=-0.31, *p* < 0.01), and thermographic measurement on the dorsoventral view correlated with breed (*r*=-0.30, *p* < 0.01). The weight distribution of both pelvic limbs correlated with joint extension (*r*=-0.36, *p* < 0.01), while considering the left pelvic limb, a higher value was observed (*r*=-0.43, *p* < 0.01). Variables considered in multiple regression statistically significantly predicted thigh girth F(5,84) = 26.33, *p* = 0.000, *R*^2^ = 0.610, with breed (*p* < 0.01), bodyweight (*p* < 0.01), and OFA hip score (*p* = 0.01) adding statistically significantly to the prediction.

**Table 1:** Mean values (± standard deviation) of overall weight, age, stance analysis (per pelvic limb and of the combination of both), thermography (ventrodorsal and lateral views), thigh girth and range of motion (extension and flexion) measurements, and by breed, sex and OFA score, of left and right pelvic limbs

	Weight	Age	Stance Analysis (individual limb)	Stance Analysis (both limbs)	Thermography (dorsoventral)	Thermography (lateral)	Thigh Girth	Joint Extension	Joint Flexion
	(kg, mean ± SD)	(yrs, mean ± SD)	(%, mean ± SD)	(%, mean ± SD)	(°, mean ± SD)	(°, mean ± SD)	(cm, mean ± SD)	(°, mean ± SD)	(°, mean ± SD)
Overall	26.7 ± 5.3	6.5 ± 2.2	18.9 ± 4.2	37.7 ± 5.7	24.9 ± 1.9	26.0 ± 2.3	30.5 ± 2.8	149.9 ± 8.4	55.9 ± 4.3
German Shepherd Dog	29.9 ± 6.3	5.7 ± 1.8	19 ± 0.6	38.4 ± 4.3	24.5 ± 1.7	25.6 ± 2.5	32.2 ± 2.7	151.3 ± 6.9	56.2 ± 3.6
Belgian Malinois Shepherd Dog	24.3 ± 4.1	6.5 ± 2.5	18.3 ± 5.6	37.6 ± 7.6	24.6 ± 1.5	27.6 ± 2.2	29.9 ± 2.4	148.6 ± 6.3	55.2 ± 5.4
Labrador Retriever	24.3 ± 2.5	8.7 ± 2.4	19.3 ± 4.1	38.5 ± 5.6	25.1 ± 1.6	26.6 ± 2.5	28.5 ± 2.3	147.8 ± 12.4	55.1 ± 3.5
Dutch Shepherd Dog	27.5 ± 3.9	5.3 ± 1.3	18.2 ± 3.5	36.4 ± 4.6	26.0 ± 2.5	26.9 ± 2.2	30.4 ± 2.0	152.0 ± 8.1	57.5 ± 4.2
Male	29.0 ± 5.4	6.2 ± 2.3	19.2 ± 5.1	38.3 ± 6.4	24.8 ± 1.9	25.9 ± 2.7	31.5 ± 2.7	150.1 ± 6.4	56.1 ± 4.3
Female	23.5 ± 2.8	6.9 ± 2.8	18.7 ± 3.2	37.2 ± 4.6	24.9 ± 1.7	26.1 ± 2.1	28.9 ± 2.1	149.6 ± 4.3	55.5 ± 4.3
Mild	27.4 ± 5.3	6.1 ± 2.1	18.9 ± 4.2	38.1 ± 4.1	24.9 ± 1.5	26.0 ± 2.1	31.2 ± 2.9	150.9 ± 7.4	55.8 ± 4.1
Moderate	25.4 ± 3.7	7.0 ± 3.4	18.4 ± 5.6	36.8 ± 6.6	25.0 ± 1.8	26.1 ± 2.5	29.7 ± 2.5	146.7 ± 11.7	56.1 ± 3.1
Severe	27.1 ± 4.9	7.6 ± 1.6	18.2 ± 1.5	36.4 ± 2.3	24.0 ± 1.7	25.5 ± 2.4	29.1 ± 2.6	144.9 ± 6.2	55.0 ± 4.3

With a cut-off of weight distribution of individual limbs set at 18%, significant variations were observed on joint extension (*p* = 0.02) and the frequency of an irregular, misshapen femoral head (*p* = 0.03). At the 20% cut-off point, besides the differences in joint extension (*p* < 0.01) and on the frequency of an irregular, misshapen femoral head (*p* = 0.02), significant variations were observed in joint flexion (*p* < 0.01) and HVAS (*p* = 0.03). For both pelvic limbs with the 36 and 40% cut-offs, significant variations were observed in joint extension (*p* < 0.01), function (*p* = 0.03), presence of CCO (*p* = 0.03 at 40), and of a misshapen femoral head (*p* = 0.02).

Absolute frequencies and percentages of radiographic findings, presented by overall, by breed, and by sex, in the ventrodorsal and frog-leg views, are outlined in Table 2. Each joint was analyzed individually, for a total of 100 joints. Considering specific radiographic signs, patients with irregular wear on the femoral head were older (*p* < 0.01), with worse weight distribution (*p* < 0.01) and CMI scores (*p* < 0.01). Animals with a flattened or shallow acetabulum, with an irregular outline, had lower weight distribution values (*p* = 0.03). Animals with CCO, on both the ventrodorsal and frog-legged views, were older (*p* < 0.01), had lower weight distribution values (*p* = 0.04), and had worse CMI scores (for all, *p* < 0.01). Those with new bone formation on the acetabulum and femoral head and neck were older (*p* < 0,01) and had worse PSS, Function, quality of life (*p* < 0.01), and PIS (*p* > 0.05) scores. Animals with a worn away angle at the cranial effective acetabular rim had lower thigh girth (*p* < 0.01) and joint flexion (*p* = 0.04). When CFHO was observable on the ventrodorsal, animals were heavier (*p* = 0.04) and had worse stiffness, function (*p* = 0.02), Gait, COI (*p* < 0.01), quality of life (*p* = 0.03) scores. The presence of CCO on the ventrodorsal was correlated with its presence on the frog-legged view (*r* = 0.51, *p* < 0.01). On the frog-legged view, the presence of CCO correlated with age (*r* = 0.47, *p* < 0.01) and joint extension (*r*=-0.51, *p* < 0.01).

**Table 2:** Overall, by breed and by sex, absolute frequencies and percentages within group of radiographic findings in the ventrodorsal and frog leg views, of hip joints. For each animal, both joints were considered, representing one hundred joints

Radiographic finding	Overall	GSD		BM		LR		DSD		Male		Female	
	Total/%	Total	%	Total	%	Total	%	Total	%	Total	%	Total	%
Irregular wear on the femoral head, making it misshapen and with a loss of its rounded appearance	95	34	100,0	30	100,0	17	85,0	16	100,0	28	46,7	20	50,0
Flattened or shallow acetabulum, with irregular outline	60	23	67,6	15	50,0	13	65,0	9	56,3	35	58,3	25	62,5
Caudolateral curvilinear osteophyte (CCO)	35	18	52,9	13	43,3	4	20,0	4	25,0	24	40,0	15	37,5
New bone formation on the acetabulum and on femoral head and neck	86	31	91,2	25	83,3	19	95,0	15	93,8	51	85,0	37	92,5
The angle formed at the cranial effective acetabular rim is worn away	77	26	76,5	23	76,7	20	100,0	12	75,0	45	75,0	34	85,0
Subchondral bone sclerosis along the cranial acetabular edge	98	34	100,0	30	100,0	20	100,0	16	100,0	60	100,0	40	100,0
Circumferential femoral head osteophyte (CFHO)	28	13	38,2	10	33,3	6	30,0	1	6,3	18	30,0	14	35,0
CCO on the Frog Leg view	33	14	41,2	12	40,0	8	40,0	5	31,3	20	33,3	17	42,5
CFHO on the Frog Leg view	88	32	94,1	25	83,3	19	95,0	16	100,0	55	91,7	35	87,5

Legend: *GSD* German Shepherd Dog, *BM* Belgian Malinois Shepherd Dog, *LR* Labrador Retriever, *DSD* Dutch Shepherd Dog

Overall scores, by breed and sex, of the considered CMI, are presented in Table 3. While no significant differences were observed between male and female animals, the opposite was observed between breeds. GSD had lower function scores than LR (*p* = 0.04), while DSD had better results when compared to other breeds with HVAS (*p* < 0.01 for GSD and *p* = 0.02 for LR), LOAD (*p* = 0.02 for GSD, and *p* = 0.02 for BM and *p* < 0.01 for LR), stiffness (*p* = 0.05 for GSD, and *p* = 0.01 for BM and LR), function (*p* < 0.01 for GSD, BM and LR), Gait (*p* < 0.01 for GSD and LR, and *p* = 0.02 for BM) and COI scores (*p* = 0.02 for GSD, and *p* < 0.02 for BM and LR). Age was the considered variable adding statistically significance (*p* < 0.01) for the prediction of PSS F(5,82) = 2.498, *p* = 0.04, PIS F(5,82) = 3.177, *p* = 0.01, *R*^2^ = 0.162, LOAD F(5,82) = 7.873, *p* < 0.01, *R*^2^ = 0.324, stiffness F(5,82) = 4.637, *p* < 0.01, *R*^2^ = 0.220, function F(5,82) = 11.160, *p* < 0.01, *R*^2^ = 0.405, gait F(5,82) = 4.074, *p* < 0.01, *R*^2^ = 0.199, QOL F(5,82) = 3.691, *p* < 0.01, *R*^2^ = 0.184 and COI F(5,82) = 6.046, *p* < 0.01, *R*^2^ = 0.269. Besides age, only the OFA hip score contributed to the prediction of PIS (*p* = 0.03). Correlation of age, joint extension, and CCO on a VD are presented in Table 4. Comparing animals at several cut-off points for PSS (scores of 4, 6, and 8), the same significant differences being observed consistently, with animals above the cut-off having worse joint extension (*p* < 0.01) and higher frequency of CCO on the ventrodorsal and frog-legged views (*p* < 0.01). When comparing the same cut-offs for PIS, at the 4 and 6 cut-offs, animals had to have a worse joint extension (*p* < 0.01) and higher frequency of CCO on the ventrodorsal and frog-legged views (*p* < 0.01). On the 8 cut-off point, the occurrence of all other radiographic signs was significantly higher (*p* < 0.01), and weight distribution on the left pelvic limb and both limbs was worse (*p* < 0.01).

**Table 3:** Median (range) for CBPI, HVAS, LOAD and COI, by breed, sex and OFA score, of different Clinical Metrology Instruments

	CBPI		HVAS		LOAD		COI				
	PIS	PSS					Stiffness	Function	Gait	QOL	Total
	(0–10)	(0–10)	(0–10)		(0–52)		(0–16)	(0–16)	(0–20)	(0–12)	(0–64)
Overall	2.9 (1.9–9.1)	2.8 (2.1-9.0)	6.2 (2.3–8.2)		10 (1–39)		3 (1–12)	2 (1–16)	4 (1–17)	3 (-12)	13 (1–54)
German Shepherd Dog	3.0 (1.8–9.4)	3.1 (1.3-9)	6.4 (2.1-8)		9 (1–39)		4 (1–11)	2 (1–11)	6 (1–17)	3 (0–4)	18 (3–50)
Belgian Malinois Shepherd Dog	2.5 (1.2-6.0)	2.4 (1.8–6.0)	7.0 (4.8–7.7)		8 (3–39)		3.5 (1–12)	1 (0–16)	3.5 (1–17)	4 (1–9)	9 (3–54)
Labrador Retriever	2.7 (1.0-8.2)	2.8 (1.5–7.8)	6.9 (4.1–7.9)		16 (4–36)		3.5 (1–10)	4.5 (0–10)	6 (1–15)	4 (1–12)	16.5 (2–47)
Dutch Shepherd Dog	2.2 (1.0-6.2)	2.0 (1.0-7.3)	7.3 (5.1–8.3)		5.5 (1–17)		2 (0–4)	0.5 (0–4)	1 (0–9)	2.5 (0–7)	5 (0–23)
Male	2.7 (1.2–8.6)	2.5 (1.3–7.3)	6.2 (4.3–8.3)		9.5 (1–39)		4 (0–12)	2 (0–16)	4.5 (1–17)	4 (0–9)	13.5 (4–54)
Female	2.3 (1.0-9.4)	2.9 (1.5-9.0)	6.1 (2.1–8.3)		10 (1–39)		1 (1–11)	3 (0–12)	4 (1–17)	3 (0–12)	11 (2–54)
Mild	6.5 (1.5–6.2)	2.1 (1.4–6.3)	6.3 (4.-8.3)		10.5 (1–36)		3 (0–12)	2.5 (0–16)	4.5 (0–17)	3.5 (0–12)	13 (0–47)
Moderate	5..0 (1.0-8.6)	5.0 (1.0-7.8)	5.7 (4.1–7.7)		16 (1–36)		4 (1–12)	4.5 (0–16)	6.6 (1–17)	5.0 (0–12)	20 (3–50)
Severe	5.0 (1.0-9.4)	5.0 (1.0–9.0)	5.7 (2.1–7.9)		23 (1–39)		5 (1–12)	6 (0–16)	8 (1–17)	6.0 (0–12)	25 (3–54)

Legend: *GSD* German Shepherd Dog, *BM* Belgian Malinois Shepherd Dog, *LR* Labrador Retriever, *DSD* Dutch Shepherd Dog, *CBPI* Canine Brief Pain Inventory, *PIS* Pain Interference Score, *PSS* Pain Severity Score, *HVAS* Hudson Visual Analogue Scale, *LOAD* Liverpool Osteoarthritis in Dogs, *COI* Canine Orthopedic Index, *QOL* Quality of Life

**Table 4:** Correlation of age, joint extension and presence of caudolateral curvilinear osteophyte (CCO) on a ventrodorsal view with different Clinical Metrology Instruments

Measure		Score							
		**PSS**	**PIS**	**LOAD**	**COI**	**Stiffness**	**Function**	**Gait**	**QOL**
Age	r_s_	0,56	-0,32	0,5	0,48	0,43	0,59	0,38	0,40
	Sig.	0,10	< 0,01*	< 0,01*	< 0,01*	< 0,01*	< 0,01*	< 0,01*	< 0,01*
Joint extension	r_s_	0,33	0,41	0,44	0,5	0,48	0,49	0,44	0,40
	Sig.	< 0,01*	< 0,01*	< 0,01*	< 0,01*	< 0,01*	< 0,01*	< 0,01*	< 0,01*
CCO	r_s_	-0,45	-0,35	0,42	0,33	-0,37	0,07	-0,36	-0,31
	Sig.	< 0,01*	< 0,01*	0,23	0,56	< 0,01*	0,12	< 0,01*	< 0,01*

Legend: *PIS* Pain Interference Score, *PSS* Pain Severity Score, *LOAD* Liverpool Osteoarthritis in Dogs, *COI* Canine Orthopedic Index, *QOL* Quality of Life. * indicates significant difference

## Discussion

Hip OA is very common in large breeds such as German Shepherd Dogs and Labrador. In working dogs, it has a toll on performance and quality of life [37, 38]. To our knowledge, this is the first study to describe the clinical presentation of Police working dogs first diagnosed with hip OA. It presents a wide variety of physical examination results and several diagnostics to provide an in-depth description of affected animals.

Radiographic examination is a staple in OA evaluation. Still, it is also well established that radiographic signs develop later than the structural changes associated with OA, and clinical symptoms do not always correlate with radiographic signs [9, 39, 40]. CFHO and CCO are considered the radiographic predictors of future OA development [9]. Animals presenting with these radiographic signs had a significantly worse clinical presentation, particularly with CCO, with animals showing worse results in all considered CMIs scores, ranging from pain to lameness level and functionality. If the presence of CCO, or other radiographic findings, influences response to treatment is still to be determined. Several differences were found between OFA grades, specifically considering pain and function scores and thermographic evaluation. The sequence of these differences may occur alongside the course of OA. From mild to moderate, structural changes occur and are detected on radiographic examination, specifically CCO, one of the predictive signs of OA development [13–15]. These structural changes are then reflected in clinical signs, such as muscular atrophy and pain, which takes a toll on daily activities. With severe OA, a corresponding loss of functional tissue and muscle masses surrounding the joint occurs [21, 41]. These facts may account for the decrease in thermographic evaluation observed in severe hip grades compared to moderate hip grades. OFA hip was also one of the variables, alongside age, adding statistically significantly to the prediction of PIS scores.

Some of the differences observed during the physical examination, as the fact that GSD were significantly heavier than other breeds (such as BM and LR), also having greater thigh muscle masses, were expected. This relation also applies to male dogs being heavier than females and with higher thigh girth. Multiple regression analysis showed the effect of breed and bodyweight in predicting thigh girth, confirming these findings. It also showed that OFA hip significantly influenced thigh girth, making it a useful measure in evaluating hip OA. These variables combined may lead to a positive correlation observed between thigh girth, weight, sex, and breed. The role that weight exerts in the development of hip dysplasia, and consequent hip OA, has been intensively studied, with heavier dogs showing to be more prone to develop OA earlier in life [42, 43]. This role is particularly true in dogs with higher body condition scores [4]. All of the animals included in this sample had either a 4 or 5 body condition score. Still, the fact that male dogs tend to be heavier than females (a tendency confirmed in this study) may place them under greater risk of developing OA and may account for the higher number of males observed. However, the OFA hip score was not predicted based on breed, age, sex, or bodyweight, so future studies should clarify these facts. Hip OA, when compared with OA in other joints, seems to be better tolerated by animals. This ability is mainly due to the higher amount of muscle masses surrounding this joint [8]. The quadriceps muscle group is particularly prone to atrophy secondary to decreased limb function. Therefore, measuring thigh girth helps make an initial assessment and measure patient evolution and treatment outcome [44]. In this study, we described thigh girth measurements of dogs initially diagnosed with hip OA, specifically of the breeds most commonly used as working and sporting dogs. However, it would be of interest to also have healthy subjects’ values to compare both groups.

The evaluation of joint ROM is a standard measurement, with OA joints usually exhibiting ROM restrictions. In the hip joint, specifically, a ROM decrease and particularly during extension, can also be present, even though this is not a universal finding [33, 39]. It showed a correlation with age, which may be attributed to disease progression since some of the older animals had worse OFA scores. Normal ROM of the hip joint for some breeds have been described. In military working GSD, a normal ROM of 44°±6 at flexion and 155°±6 at extension, and in LR of 50°±2 at flexion and 162°±3 at extension have been reported [45–47]. Our study measured lower values in both breeds, which could be expected due to OA. Still, it would be interesting to have a group of disease-free dogs to compare these values and describe normal values in the other two considered breeds.

The mean age of animals included in this sample was 6.5 years, which is earlier than the commonly considered risk factor for OA of > 8 years [4]. GSD and DSD were even younger than 6.5 years, with only LR being beyond this point and significantly older than the other breeds. Multiple regression analysis showed that age was the primary variable adding statistically significantly to CMI scores’ prediction. All of the animals included in the sample were screened before starting training and active work, so the earlier diagnosis may be attributed to the high demand and stress that these animals’ musculoskeletal structures are under and the subsequent toll on performance [48]. Since these animals are active working dogs, it is possible that the disease actually develops or is simply detected earlier than in other dogs. The reason leading to a later diagnosis of LR is not clear. It may be due to breed characteristics, with LR being less explosive and less driven than BM, for example. Also, a less physically demanding mission of these dogs (most were product detection dogs) compared with the remaining animals included in the sample (mostly involved in search and rescue and use of force activities) might be an important factor to consider.

Normal weight distribution on the weight distribution plate is the same as for pressure-sensitive walkway total pressure index—30/30/20/20 (left thoracic limb/right thoracic limb/left pelvic limb/right pelvic limb) [49, 50]. For the evolution of hip OA, bodyweight distribution at a stance may even be a superior measurement to VI and PVF since dogs present different standing postures to increase acetabular coverage. Sensitivity and specificity seem to be higher with a cut-off point of 18% for pelvic limbs [8, 18, 51]. We considered both the 20% and 18% cut-off, with more differences being found at 20%. Mean values were below the 20% value but showed some dispersion. Since included animals had bilateral disease, it is quite possible that at any given point, they would be overloading one side to protect the other, leading to very different weight distribution values when comparing contralateral limbs in the same animal. Dogs presenting with pelvic limb-lameness tend to distribute weight more side-to-side than pelvic-to-thoracic compensation [52, 53]. For that reason, we also analyzed weight distribution for both pelvic limbs, with two different cut-off points. This analysis may be an interesting approach since it accounted for significant joint extension and function scores and CCO variations. It would be interesting to see the importance of these cut-off points in evaluating response to treatment. It should be the subject of further research, mainly since it did not show associated breed variations. It has been described that male dogs tend to carry more weight on the thoracic limbs naturally and may exhibit fewer improvements in response to treatment [17]. No significant variation comparing males and females in weight distribution was found, but future studies should evaluate this hypothesis.

Canine thermal imaging has been documented only recently. Still, a growing interest in this modality has led to an increase in the number of studies evaluating its use to assess the canine hip, stifle, elbow, and intervertebral disc [24, 54–58]. To our knowledge, this is the first study describing values for dogs with hip OA. The coat’s type and color are variables that must be taken into account, and its influence documented [55, 56, 59, 60]. Our results seem to confirm this fact since DSD showed significantly different values than other breeds, and this may be due to its brindle coat, in opposition to lighter coats in the other breeds. In humans OA studies, increased temperatures have been related to even slight degenerative changes and low temperatures in more severe disease cases [61]. In this study, this effect was not found, but it may be due to the coat variation effect. Still, its value in evaluating response to treatment has to be determined.

CMIs represent a patient-centred approach that, similar to what happens in human medicine, has been incorporated in veterinary assessments in different species [62–64]. They may also capture a different dimension of OA since owners may often be more focused on the dog’s ability to perform daily activities, rather than an increase or decrease of ROM or use of a single limb at a walk or trot [65, 66]. While no differences were observed when comparing animals by sex, several differences were observed between breeds and reported values for the same breeds’ pet dogs. One of the reasons for this may be the nature of the specific mission of the animals. When involved in a more physically challenging task, it is more likely that complaints or limitations arise. Another reason may be age (which correlated with several scores), since older animals tend to be more experienced and able to manage the effort, making them less prone to injury [67]. Also, since these animals are selected based on working predisposition, they present high drive, which may mask some complaints and lead, for example, to relatively low PSS. We also aimed to see if different cut-off points of pain scores (measure with the PIS and PSS) presented significant differences. The main finding was that, as could be expected, animals with higher PIS scores had significantly lower weight distribution, but also had higher frequencies of all radiographic signs.

This study presents some limitations, namely the lack of a control group with non-lame dogs. This limitation is mainly related to the sample’s convenience nature, comprised of dogs specifically presenting for treatment. Some of the previous report results of similar evaluations were conducted in the same breeds included in our sample, which is still useful. Since data was only collected in a single moment, we cannot comment on the interest of each of the findings for the prognosis or treatment monitoring of OA, which should be addressed in future studies.

## Conclusions

To our knowledge, this study first describes several clinical and radiographic findings of working dogs of different breeds to hip OA. Police working dogs presented complaints related to hip OA at an early stage of the disease and a younger age than non-working dogs. LR were significantly older than other considered breeds. Hip scores influenced clinical presentation, with moderate cases showing lower thigh girth and worse PIS, PSS, and function scores than mild cases. Patients with severe OA had lower thermographic evaluations than patients with moderate OA. Age was the primary variable influencing considered CMI scores.

## Methods

The sample comprised fifty (N = 50) Police working dogs with bilateral hip OA. It was a convenience sample, composed of patients presented at the Clínica Veterinária de Cães (Portuguese Gendarmerie Canine Clinic) to undergo hip OA treatment after initial diagnosis. Subsequent treatment was randomly determined, as the animals took part in a study evaluating intra-articular treatments for OA. Patients were active police working dogs of the Guarda Nacional Republicana (Portuguese Gendarmerie Canine Unit). The diagnosis was based on the dog’s history, trainer complaints (difficulty rising, jumping and maintaining obedience positions, stiffness and decreased overall performance), physical examination (pain during joint mobilization, stiffness and reduced range of motion), and radiographic findings (OFA hip scores of mild, moderate or severe) consistent with bilateral hip OA. Inclusion criteria were: bodyweight ≥ 15 kg, animal older than 2 years and without any medication or nutritional supplements for 6 weeks or more before the beginning of the study. Animals suspected or with any other orthopaedic or concomitant disease (ruled out through physical examination, complete blood count, and serum chemistry profile) and not tolerant of data collection were excluded. All evaluations were performed at the same moment by the same researcher, which had extensive experience in the conduction of all procedures to reduce inter-observer variability.

### Digital thermography

**Fig. 1:**
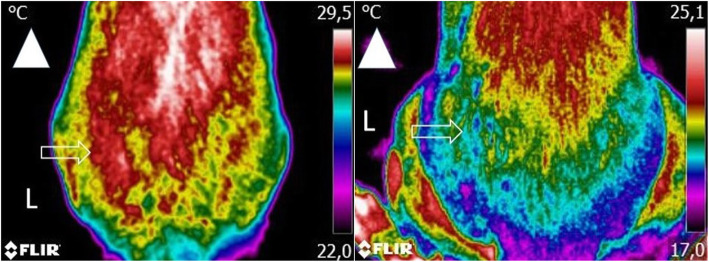
A dorsoventral view of a dog with moderate osteoarthritis (left) and another with severe osteoarthritis (right), including the area from the last lumbar vertebra to the first coccygeal vertebra at a minimum, at a distance of 60 cm. Arrowhead indicates cranial direction. Arrow indicates the anatomical location of the hip joint. An area of increased temperature is observed on the patient with moderate OA and of lower temperature on the patient with severe OA

For the collection of digital thermography images, dogs were allowed to walk around in a large, plain wall room and adjust to room temperature (set at 21 °C) in a relaxed way for approximately 30 min before imaging. They were then positioned in an upright standing position, as symmetrically as possible, without the trainer or veterinarian touching its torso. A dorsoventral and two lateral images (one for each limb) were obtained from every animal. Every dorsoventral thermographic image included the last lumbar vertebra area to the first coccygeal vertebra at a minimum, at a distance of 60 cm (Fig. 1) [23]. Lateral views had the greater trochanter in the centre of the image, also at a distance of 60 cm. All images were captured with a FLIR ThermaCAM E25® camera model and kept when the anatomical landmarks were included, and the image was steady enough to determine their location. The free software Tools (FLIR Systems, Inc) was used to analyse the images, with a rainbow color pallet. Temperature boxes of equal size were placed on the hip joint’s anatomical area on both views, with mean and maximal temperatures determined.

### Stance Analysis

Stance analysis was conducted with a weight distribution platform (Companion Stance Analyzer; LiteCure LLC®, Newark, Delaware, United States). According to the manufacturer’s guidelines, it was placed in the centre of a room, at least 1 meter from the walls. It was calibrated at the beginning of each day, and zeroed before each data collection. Animals were encouraged to stand on to the weight distribution platform. Its trainer helped ensure the patients placed one foot on each quadrant of the platform while maintaining a natural stance with the centre of gravity and stability (measured by the platform) near the platform’s middle. Gentle restraint was used to keep the patient’s head in a natural, forward-facing position when needed. For all animals, at least 20 measurements were performed, and the mean value was determined. Normal weight distribution for each pelvic limb was considered 20% of the total weight [18]. Since all animals included had bilateral OA, weight distribution on both pelvic limbs was also considered and set at 40% (20% left pelvic limb + 20% right pelvic limb).

### Clinical Assessment

Determination of thigh girth was made with a Gullick II measuring tape at a distance of 70% thigh length, measured from the tip of the greater trochanter, with the leg in an extended position while in lateral recumbency, and the dog relaxed [44]. With the patient in the same position, hip joint ROM was obtained with a goniometer (Veterinary Instrumentation, United Kingdom) at extension and flexion, with a flexed stifle [68]. These measurements were made in triplicate, and the mean value was calculated.

### Radiographic examination

Radiographic studies were conducted under light sedation, using a combination of medetomidine (0.01 mg/kg) and butorphanol (0.1 mg/kg), given intravenously. A ventrodorsal extended legs view and a frog-legged view were obtained. Hips were graded according to the OFA hip grading scoring scheme[69] by the researcher, blinded to the patient’s identification. A mild score corresponded to a partially subluxated femoral head, causing an incongruent and widened joint space, with a shallow acetabulum, only partially covering the femoral head. In young dogs (24 to 36 months), OA lesions may not be present. Moderate grades were attributed when significant subluxation was present, and the femoral head was barely seated into a shallow acetabulum. Secondary remodeling along the femoral neck and head, acetabular osteophytes, and subchondral sclerosis were present. In severe cases, the femoral head was partly or completely out of a shallow acetabulum, with extensive secondary arthritic bone changes along the femoral head and neck head, acetabular rim changes, and large amounts of abnormal bone pattern changes. A full description of the OFA hip grading scheme is available online (https://www.ofa.org/diseases/hip-dysplasia/grades). The presence of specific radiographic signs was also recorded: irregular wear on the femoral head, making it misshapen and with a loss of its rounded appearance; a flattened or shallow acetabulum, with irregular outline; CCO; new bone formation on the acetabulum and femoral head and neck; a worn away angle formed at the cranial effective acetabular rim; subchondral bone sclerosis along the cranial acetabular edge; and CFHO [9, 39, 70, 71]. In the frog-legged view, the presence of CCO and CFHO was also recorded.

### Clinical metrology instruments

At the evaluation moment, an online copy prepared for the effect of the HVAS, CBPI, COI, and LOAD was completed by the trainers. The same trainer completed all CMIs for each dog.

### Statistical Analysis

Normality was assessed with a Shapiro-Wilk test. Each measured parameter was compared with an Independent Samples T-Test (when two groups were considered, like sex) or ANOVA, followed by a Bonferroni post hoc test for multiple comparisons (when more than two groups were considered). CMI scores were compared with a Wilcoxon signed-rank test. Different score cut-off points (4, 6, and 8) were analyzed for PIS and PSS. 20% and 18%[18] pelvic limb percentages cut-off points were considered for weight distribution. Since hip OA is often bilateral, results for the combination of both pelvic limbs were also analyzed, at 36% (18% left pelvic limb + 18% right pelvic limb) and 40% (20% left pelvic limb + 20% right pelvic limb). The correlation between parameters was assessed with the Pearson correlation coefficient. Multiple regression was run to predict evaluated parameters from age, sex, breed, body weight, and OFA hip score. All results were analyzed with IBM SPSS Statistics version 20, and a significance level of *p* < 0.05 was set.

## Data Availability

The data that support the findings of this study are available from the Guarda Nacional Republicana (Portuguese Gendarmerie) but restrictions apply to the availability of these data, which were used under license for the current study, and so are not publicly available. Data are however available from the authors upon reasonable request and with permission of [the Divisão de Medicina Veterinária of the Guarda Nacional Republicana.

## Abbreviations

BM: Belgian Malinois
CBPI: Canine Brief Pain Inventory
CCO: Caudolateral curvilinear osteophyte
CFHO: Circumferential femoral head osteophyte
COI: Canine Orthopeadic Index
DSD: Dutch Shepherd Dog
FL: Frog-leg view
GSD: German Shepherd Dogs
HVAS: Hudson Visual Analogue Scale
LOAD: Liverpool Osteoarthritis in Dogs
LR: Labrador Retriever
OA: Osteoarthritis
PIS: Pain Interference Score
PSS: Pain Severity Score
ROM: Range of motion
VD: Ventrodorsal view
